# Agathisflavone Modulates Reactive Gliosis After Trauma and Increases the Neuroblast Population at the Subventricular Zone

**DOI:** 10.3390/nu16234053

**Published:** 2024-11-26

**Authors:** Juliana Helena Castro e Silva, Francesca Pieropan, Andrea Domenico Rivera, Arthur Morgan Butt, Silvia Lima Costa

**Affiliations:** 1 Laboratory of Neurochemistry and Cellular Biology, Department of Biofunction, Health Sciences Institute, Federal University of Bahia, Salvador 40231-300, Brazil; juli.hele@gmail.com; 2 School of Medicine, Pharmacy and Biomedical Sciences, University of Portsmouth, Portsmouth PO1 2UP, UK; fpieropan@gmail.com (F.P.); riveraandrea83@yahoo.it (A.D.R.); 3 Southampton Solent University, E Park Terrace, Southampton SO14 0YN, UK

**Keywords:** flavonoid, agathisflavone, traumatic brain injury, glial scar, neuroinflammation, neurogenesis

## Abstract

Background: Reactive astrogliosis and microgliosis are coordinated responses to CNS insults and are pathological hallmarks of traumatic brain injury (TBI). In these conditions, persistent reactive gliosis can impede tissue repopulation and limit neurogenesis. Thus, modulating this phenomenon has been increasingly recognized as potential therapeutic approach. Methods: In this study, we investigated the potential of the flavonoid agathisflavone to modulate astroglial and microglial injury responses and promote neurogenesis in the subventricular zone (SVZ) neurogenic niche. Agathisflavone, or the vehicle in controls, was administered directly into the lateral ventricles in postnatal day (P)8-10 mice by twice daily intracerebroventricular (ICV) injections for 3 days, and brains were examined at P11. Results: In the controls, ICV injection caused glial reactivity along the needle track, characterised immunohistochemically by increased astrocyte expression of glial fibrillary protein (GFAP) and the number of Iba-1+ microglia at the lesion site. Treatment with agathisflavone decreased GFAP expression, reduced both astrocyte reactivity and the number of Iba-1^+^ microglia at the core of the lesion site and the penumbra, and induced a 2-fold increase on the ratio of anti-inflammatory CD206+ to pro-inflammatory CD16/32+ microglia. Notably, agathisflavone increased the population of neuroblasts (GFAP+ type B cells) in all SVZ microdomains by up to double, without significantly increasing the number of neuronal progenitors (DCX+). Conclusions: Although future studies should investigate the underlying molecular mechanisms driving agathisflavone effects on microglial polarization and neurogenesis at different timepoints, these data indicate that agathisflavone could be a potential adjuvant treatment for TBI or central nervous system disorders that have reactive gliosis as a common feature.

## 1. Introduction

Traumatic brain injury (TBI) is one of the most prevalent causes of central nervous system (CNS) disease. It is estimated to affect approximately 54 to 60 million individuals every year, leading to disabilities, death, and causing an impactful social and economic burden [1]. As a result of external mechanical forces in the head, a complex cascade of events contributes to disability. In the more acute phase of the injury, axons get damaged, and the blood–brain barrier is disrupted, causing blood cells to migrate into the CNS [2]. The second phase after the injury is characterized by a neuroinflammatory CNS cellular response that generate neuronal excitotoxicity and oxidative stress. At this stage, glial cells populate the damaged areas in a co-ordinated response known as reactive gliosis. This response is triggered also after other ischemic and neuroinflammatory injuries and is often referred to as ‘glial scarring’, which can be misleading because the scar tissue is mainly formed by perivascular fibroblasts, whereas transformed astrocytes form a perilesional barrier, termed the glia limitans perilaesiones [1,3,4,5,6]. At this phase, astrocytes undergo hypertrophy and upregulate the expression of filamentous proteins such as the glial fibrillary acidic protein (GFAP) and vimentin, forming a physical barrier at the injury site. This limits the spreading of blood-derived cells to the rest of the CNS [7]. Together with oligodendrocyte precursor cells, they reshape extracellular matrix composition and provide metabolic support for axon regrowth [8]. Microglia remove cell debris and, together with astrocytes, trigger the inflammatory response that can reestablish physical integrity of the nervous tissue and resolve the injury [9,10]. However, the prolongation of reactive gliosis can lead to detrimental extracellular matrix composition and exacerbated inflammatory response that impede axon regrowth and tissue repopulation by neurons, which hinders full CNS recovery [8,11,12].

Effective management of TBI involves an integrative approach that combines acute medical intervention, neurorehabilitation, and long-term supportive care. The pharmacological treatments mostly aim at managing neuropsychological problems right after the damage. On the other hand, there are very limited available pharmacological options that help to restrain CNS damage by preventing bleeding and inflammation and depressing brain activity, such as tranexamic acid, corticosteroids, and sedatives, among others [13].

In this context of prolonged neuroinflammation and limited neurogenesis and tissue repopulation, modulating reactive gliosis and motivating neurogenesis have both been proposed as novel potential therapeutical approaches to CNS trauma [14].

Agathisflavone (6,8″-bisapigenin, C_30_H_18_O_10_) is a biflavonoid constituted by two molecules of apigenin bound by carbons 6 and 8 of the aromatic ring ‘A’ [15]. Many biological activities of agathisflavone are described in the literature [16]. Particularly, we have previously demonstrated that agathisflavone (FAB) reduces astrogliosis and modulates microglial activation in in vitro and ex vivo models of CNS trauma, neuroinflammation, and demyelination [17,18,19,20,21]. In an in vitro model of TBI using a scratch wound induced by a pipette tip, agathisflavone (1 μM) reduced astroglial GFAP expression and hypertrophy while promoting an increase in neuronal numbers and neurite outgrowth into the lesion site. Additionally, it enhanced the expression of neurotrophic factors NGF and GDNF, which are linked to a neuroprotective glial cell profile. This finding suggests that FAB modulates the astrocytic response to injury and glial scar formation, which can foster neural regeneration [17].

Additionally, agathisflavone was shown to induce neurogenesis and neuronal maturation in vitro, and improved tissue repair potential of mesenchymal cell injection in spinal cord injury in vivo [22]. A study using mouse embryonic stem cell cultures evaluated the potential of agathisflavone to induce neuronal differentiation in aggregates of these cells, in isolated treatments and/or combined with retinoic acid (RA). Treatments with agathisflavone (60 μM) resulted in a significant increase in the number of Nestin+ cells, a marker of neural progenitors, and β-tubulin III+ cells, a marker of young neurons. A similar effect was observed in induced pluripotent stem cells, with an increase in Nestin+ and β-tubulin III+ cells by sustaining neural progenitors apoptosis rate [23]. In a co-culture of neurons and glia, treatments with agathisflavone (10 μM) for 72 showed an increase in the expression of doublecortin+ cells (young and migratory neurons), β-tubulin III+ cells (young neurons), MAP2+ cells (post-mitotic neurons), and vGlut2+ cells (glutamatergic neurons), without any change in the number of microglia and astrocytes, but with an increased expression of neurotrophins NGF, BDNF, GDNF, and NT4 after glutamate excitotoxicity [21]. Furthermore, agathisflavone also rescued the expression of pro-inflammatory cytokine genes in this model [21]. FAB also has demonstrated neuroprotective activities by modulating microglial phenotype toward a more regenerative profile and by reducing the expression of pro-inflammatory cytokines in a model of demyelination induced by lysophosphatidylcholine in organotypic cerebella slices [20], which supports that FAB could be an adjuvant in therapy by acting as anti-neuroinflammatory and targeting reactive gliosis.

Thus, taking the neuroprotective effects of FAB into consideration, we aimed to evaluate the in vivo effects of FAB on reactive gliosis following a forebrain stab wound induced by intracerebroventricular (ICV) injection into the lateral ventricles. The results show that agathisflavone reduces reactive gliosis and promotes neurogenesis at the subventricular zone (SVZ), one of the two neurogenic niches in the adult brain, supporting a potential neuroprotective effect of FAB treatment in TBI.

## 2. Materials and Methods

### 2.1. Agathisflavone Extraction

The biflavonoid agathisflavone (6,8″-Biapigenin; FAB) was obtained from *Poincianella pyramidalis* (Tul.) L.P.Queiroz (syn. *Cenostigma pyramidale* (Tul.) Gagnon & G.P. Lewis) leaves according to Mendes et al. [15], with 99% of purity. Stock solutions were prepared at 10 mM concentration in dimethyl sulfoxide (DMSO; Sigma Chemical Co., St. Louis, MO, USA) and stocked at −4 °C. Before injections, FAB was diluted to 100 μM in sterile phosphate-buffered saline (PBS) (FAB). The control group was treated with PBS containing the same amount of DMSO in sterile PBS (vehicle, Veh).

### 2.2. Animals and Treatments

The C57/BL6 wild-type or transgenic mice in which the expression of the enhanced green fluorescent protein (eGPF) is driven by the human glial fibrillary acidic protein (hGFAP) were used (TgN(GFAP-EGFP)GFEC-FKi line). Transgenic mice were obtained at the University of Saarland, Germany and gifted by Professor Frank Kirchhoff [24]. All procedures were performed according to the UK Home Office Animals Scientific Procedures Act (1984) under the license P9378105 (24 March 2023). At the eighth postnatal day, animals were randomly selected and deeply anesthetized with isoflurane and injected in the SVZ as previously described [25,26]. Briefly, mice were injected intraventricularly twice a day for three days with two microliters of FAB or Veh. A total of 14 mice of unspecified sex were used, 6 wild-type and 8 transgenic, equally distributed between Vehicle- and FAB-treated. One animal per group was excluded for the first lesion analyses because there was no visible lesion in their cortices. Twenty-four hours after the last injection, mice were deeply anesthetized and sacrificed by cervical dislocation. The entire brain was dissected and fixed in paraformaldehyde (PFA, Sigma Chemical Co., St. Louis, MO, USA) 4% for 24 h, then washed with PBS.

### 2.3. Immunostaining

For immunostaining, brain tissues were sliced coronally with a vibratome (Leica VT1000S, Wetzlar, Germany) at 50 μm thickness and free-floating slices were stored in 0.05% sodium azide-containing PBS. Three slices per animal containing the sub-ventricular zone (SVZ) and visible cortical scars were selected. For immunohistochemistry, two protocols were used. Slices were submitted to sodium borohydride (Sigma Chemical Co., St. Louis, MO, USA) 1% permeabilization for 30 min followed by phosphate buffer washings and blocking in 1% normal goat serum (NGS, Invitrogen, Waltham, MA, US) and 0.5% Bovine Serum Albumin (BSA; Sigma Chemical Co., St. Louis, MO, USA) for 2 h at room temperature (RT) and agitation. Slices were washed in Tris-buffered saline (TBS) and incubated in an NGS-TBS-Triton X-100 0.25% antibody solution containing anti-Ki67 (Invitrogen, Waltham, MA, USA 145698; Rat; 1:500) overnight at RT and agitation. Following incubation, sections were washed in PBS–Triton 0.25% and incubated with corresponding Alexa Fluor secondary antibodies (Invitrogen, Waltham, MA, USA; Goat; 1:500) and Höechst 33258 (1:1000, Invitrogen, Waltham, MA, USA) for 3 h at room temperature with agitation, and then washed and mounted.

For GFAP, doublecortin and microglial markers Iba-1, CD206, and CD16/32, slices were washed with PBS and incubated in a blocking solution containing 10% donkey or goat serum, 1% BSA and 2% Triton X-100 in PBS for 2 h under room temperature and agitation. Slices were then immersed in an antibody solution containing 10% serum, 1% BSA and 1% Triton-X, and the following primary antibodies: anti-Doublecortin (Abcam ab18723, Cambridge, UK; Rabbit; 1:100); anti-GFAP (Millipore ab5541, Burlington, MA, USA; Chicken; 1:500); anti-CD206 (R&D Systems, Minneapolis, MI, USA; Goat; 1:400); anti-CD16/32 (Biosciences 533142, Princeton, NJ, USA; Rat; 1:400); anti-Iba-1 (Rabbit; 1:1000). The slices were incubated overnight at 4 °C. On the following day, sections were washed in PBS and incubated in secondary antibody solutions containing corresponding Alexa Fluor conjugated antibodies (Invitrogen, Waltham, MA, USA; 1:500).

### 2.4. Imaging and Analysis

Images from the sections were captured using confocal microscopy (Zeiss 710 and Zeiss LSM Image Examiner Software V. 5.2.0.121). The Z-stacks were taken at 20× magnification and images measured 780.49 × 780.49 μm in the x-y-plane, and 25 μm in the z-plane. For SVZ analysis, one slice per animal was selected aiming to have similar ventricle position and size. The SVZ was analysed at the following 3 different areas of interest: dorsal (d-SVZ), lateral (l-SVZ) and horn (h-SVZ). Counting was performed in a field of view (FOV) of 100 × 100 μm in the x-y-plane, and 25 μm in the z-plane using Fiji Software (https://imagej.net/software/fiji/, accessed on 15 August 2024) cell count tool [27].

For lesion analysis, FOVs measuring 200 × 200 μm and 25 μm in the z-plane containing cortical lesions were selected and GFAP and Höechst 33258 relative density was measured using Fiji. The same threshold was set to all images.

### 2.5. Statistics

Statistics were performed using Graphpad Prism 9.0.0 for Windows (Boston, MA, USA). All data are presented as mean ± standard deviation. Unpaired bilateral student’s *t*-test, assuming normal distribution for all datasets, was applied for comparisons between Veh and FAB groups and *p* values equal to or below 0.05 were considered statistically significant.

## 3. Results

**Figure 1:**
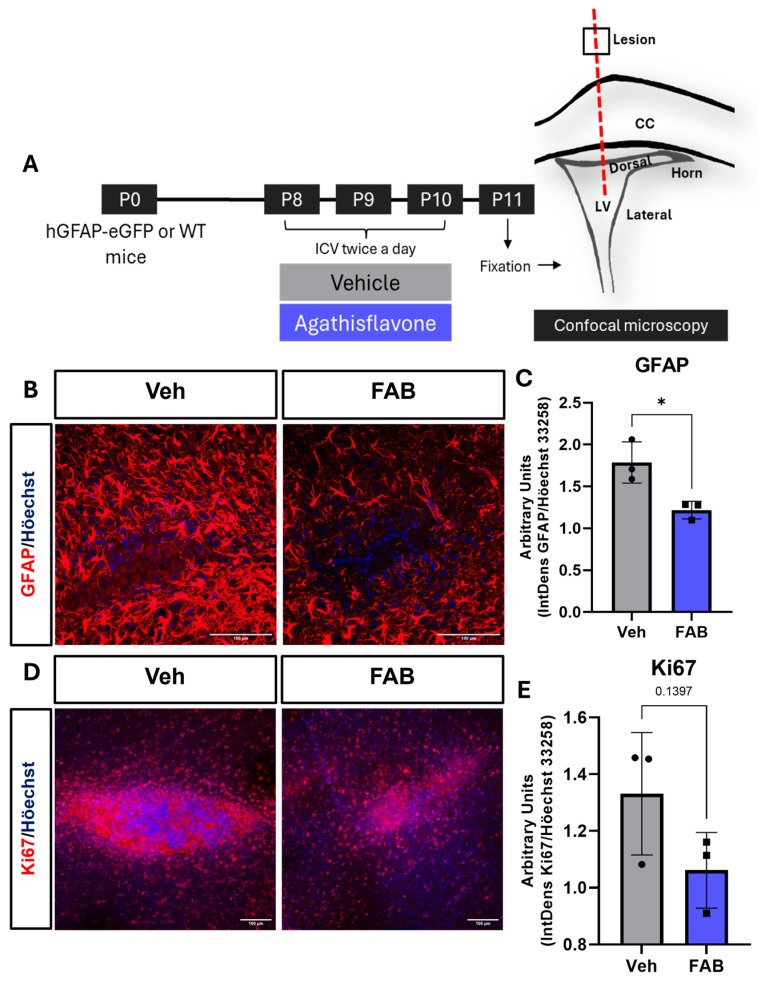
Agathisflavone decreases astrogliosis and cell proliferation following intracerebral stab wound. (**A**) A cerebral stab wound injury (SWI, red dashed line) was performed in P8-10 mice with a 26-gauge needle that was used to deliver 2 μL of sterile saline vehicle (Veh) or 100 μM agathisflavone (FAB). Injections were performed twice daily for 3 days from P8-10 and the cerebrum was collected on P11, twenty-four hours after the last injection. (**B**,**C**) Representative confocal images of GFAP immunostaining of astrocytes (red) at the lesion site following treatment with Veh (left colum) or FAB (right column), as indicated (Scale bar: 100 μm), together with quantification of GFAP expression. (**D**,**E**) Representative confocal images of Ki67 immunostaining of proliferating cells (red) at the lesion site (Scale bar: 100 μm), together with quantification of Ki67 expression. Results in (**C**,**E**) are expressed in Arbitrary Units (integrated density ratio of GFAP or Ki67 divided by Höechst 33258 nuclear staining); data are mean ± standard deviation (n = 3, dots (Veh) or squares (FAB) on column graphs) and were tested for statistical significance using unpaired *t*-tests, * *p* ≤ 0.05 in (**C**) and number over the bar is the *p* value in (**E**).

The in vivo effects of agathisflavone (FAB) on reactive gliosis and SVZ neurogenesis were investigated in mice aged postnatal day (P)8-10 following cerebral stab wound injury with a 26-gauge needle used for injection of sterile saline vehicle (Veh) or FAB. Injections (2 μL) were performed into the lateral ventricle twice a day from P8 to P10 and mice were killed on P11 twenty-four hours after the final treatment. Brains were immersion-fixed and vibratome sections were taken in the coronal plane in the region of the stab wound and the SVZ (Figure 1A). Wild-type C57/BL6 mice were used for immunohistochemical analyses of astrocytes (GFAP) and microglia (Iba1, CD16/32, CD206). For SVZ neurogenesis, GFAP-expressing type-B neural stem cells (NSC) were identified using GFAP–eGFP mice (GFAP drives expression of enhanced green fluorescent protein, eGFP) and type C (intermediate progenitors, transit amplifying cells); type C transit amplifying cells and type A neuroblasts were distinguished by immunostaining for doublecortin (DCX) in combination with the cell proliferation marker, Ki67.

### Agathisflavone Modulates Reactive Gliosis in the Mouse Lesioned Cortex

The cerebral stab wound injury caused visible astrocyte reactivity at the lesion site, evidenced by a prominent increase in GFAP immunostaining with apparent astrocyte hypertrophy, and quantitative analysis of GFAP immunostaining was significantly reduced by treatment with FAB (Figure 1B,C); the integrated density ratio of GFAP immunofluorescence was measured relative to Höechst 33258 nuclear staining in a 200 × 200 μm field of view, FOV). Immunostaining for Ki-67, a nuclear protein that is found only in dividing cells, indicated marked cellular proliferation at the lesion site in controls, consistent with reactive gliosis, and this was decreased by FAB treatment, although the mean effect was not statistically significant (Figure 1D,E).

**Figure 2:**
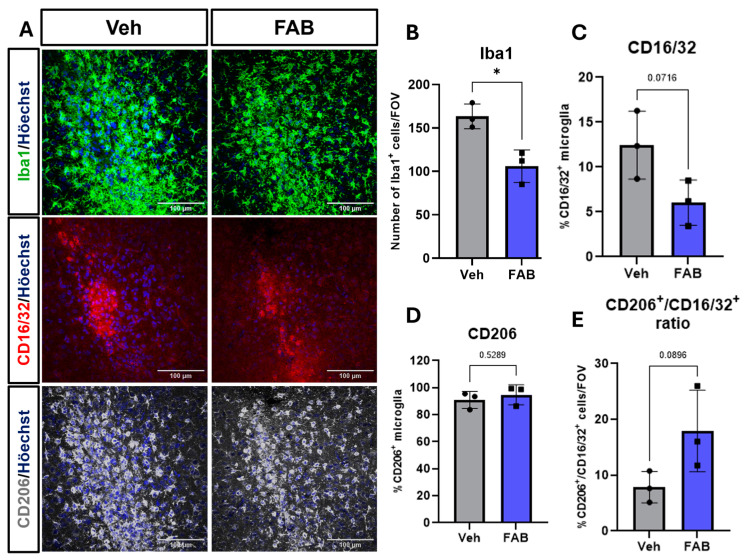
Agathisflavone decreases microglia number and modulates microglial activation phenotype at the lesion site. (**A**) Representative confocal images of coronal sections following a cerebral stab wound injury induced by a 26-gauge needle that was used to deliver 2 μL of sterile saline vehicle (Veh) or 100 μM agathisflavone (FAB). Sections were immunostained for the general microglial marker Iba1 (green, upper panels) and markers of activated microglia CD16/32 (red, middle panels) and CD206 (grey, lower panels; scale bars = 100 μm). (**B**) Quantification of cellular density of Iba1-positive cells at the lesion site in a 200 × 200 μm field of view (FOV). (**C**,**D**) Quantification of the density of Iba1+ cells co-expressing CD16/32 and CD206. (**E**) Ratio of CD206+ to CD16/3+ microglia in the same field of view. Data are expressed in mean ± standard deviation (n = 3) and were tested for significance using unpaired *t*-tests, * *p* ≤ 0.05.

Next, we investigated the microglial reaction to the cerebral stab wound. In controls, immunostaining for Iba1 indicated a marked microgliosis at the lesion site and this was significantly reduced by treatment with FAB, which resulted in an approximately 40% reduction in the number of Iba1+ microglia compared to the controls (Figure 2A,B). Particular microglial activation profiles are related to injury and repair, so we also investigated the expression of CD16/32 (Fcγ receptors), a marker for pro-inflammatory and phagocytic microglia, and CD206 (also known as mannose receptor C type 1, MRC1), which are associated with anti-inflammatory-activated microglial and improved recovery from traumatic brain injury [28]. There is clear evidence of both CD16/32+ and CD206+ cells at the lesion site (Figure 2A); CD206 immunostaining mirrored that for Iba1 and was most dense within the brain tissue bordering the lesion, indicative of activated microglia, whereas CD16/32+ cells were localized to the lesion core, suggesting many of these cells may be vascular macrophages. Quantification of CD16/32+ and CD206+ cells as a percentage of Iba1+ cells confirmed that the majority of activated microglia had a CD206+ phenotype and these far outnumbered CD16/32+ cells by a factor of over 7:1 (Figure 2B–D), indicating the majority of activated microglia had an anti-inflammatory phenotype associated with tissue repair. Treatment with FAB had no significant effect on the relative densities of CD16/32+ or CD206+ microglia (Figure 2C,D), but did have a significant 2-fold effect on the ratio of CD206+ to CD16/3+ cells (Figure 2E), suggesting FAB shifted microglial activation to a more repair-oriented phenotype.

**Figure 3:**
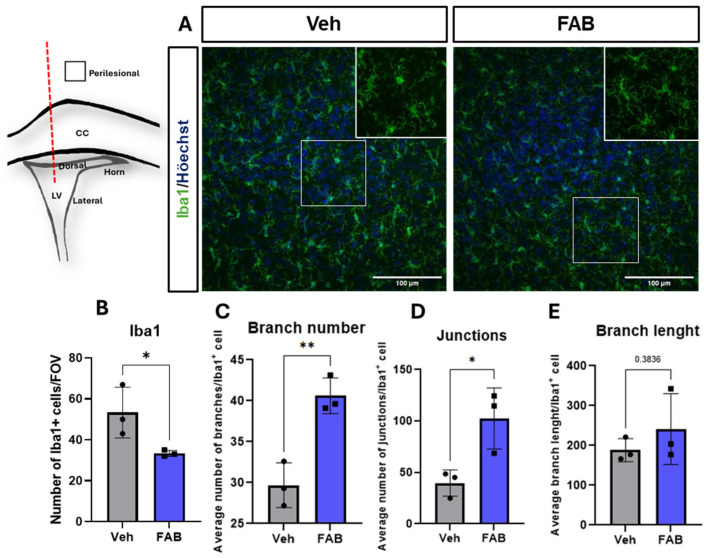
Agathisflavone modulates microglial density and morphological phenotype in perilesional sites. (**A**) Representative confocal images of perilesional sites following a cerebral stab wound injury (SWI, traced line on left drawing) induced by a 26-gauge needle that was used to deliver 2 μL of sterile saline vehicle (Veh) or 100 μM agathisflavone (FAB). Sections were immunostained for the general microglial marker Iba1 (green); scale bars = 100 μm). (**B**) Quantification of the density of Iba1+ microglia in perilesional sites. (**C**–**E**) Morphological analysis of the processes of Iba1+ microglia using Fiji application (https://imagej.net/software/fiji/, accessed on 15 August 2024) and skeleton analysis to quantify the number of process branches (**C**), junctions (**D**), and branch length (**E**), normalized by the number of Iba1-positive cells in the same field of view. Data are expressed as mean ± standard deviation (n = 3) and tested for statistical significance using unpaired *t*-tests, * *p* ≤ 0.05, ** *p* ≤ 0.005.

The effects of FAB on microglia at the lesion site prompted us to examine microglial activation at perilesional sites, where microglia were less dense, and it was possible to analyse the morphology of Iba1+ microglia (Figure 3). In line with the effects of FAB at the lesion site, FAB also induced a significant decrease in the number of perilesional Iba1+ microglia compared to controls (Figure 3B); the decrease in microglia was proportionally similar to that at the lesion site, indicating effective diffusion of FAB within through the brain tissue. In addition, FAB treatment had a significant effect on microglial morphology in perilesional sites, with significantly increased number of branches and branch junctions along cellular processes (Figure 3C,D), compared to vehicle-treated mice; no significant changes were observed in the length of microglial processes (Figure 3E). These results indicate FAB modulates microglial process arborization towards a more ramified morphology typical of a less activated or ‘resting’ microglial phenotype in brain tissue surrounding the lesion site.

**Figure 4:**
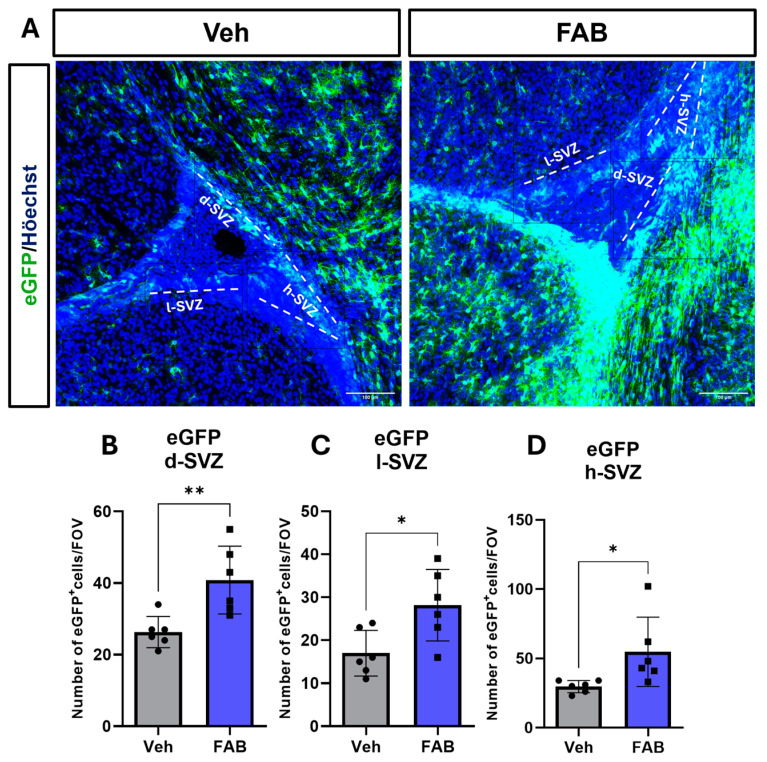
Agathisflavone increases the number of SVZ neural progenitors. (**A**) Representative confocal images of the SVZ in GFAP–eGFP mice following intracerebroventricular (ICV) injection of saline vehicle (Veh) or 100 μM agathisflavone (FAB); scale bars = 100 μm). (**B**–**D**). Quantification of GFAP–eGFP+ neural stem cells in microdomains of the dorsal SVZ (B), lateral SVZ (**C**), and the horn of the SVZ (**D**). Data are cell density per constant area expressed as mean ± standard deviation and were analysed for statistical significance using unpaired *t*-tests; * *p* ≤ 0.05, ** *p* ≤ 0.005.

We then examined whether FAB has a neurogenic effect on neural stem cells in the SVZ, the main neurogenic niche, by quantifying the number of GFAP–eGFP+ neural stem cells (NSC) in different microdomains of the SVZ, namely the dorsal, horn, and lateral SVZ (d-SVZ, h-SVZ, and l-SVZ, respectively). We observed that FAB treatment significantly increased NSC throughout the SVZ compared to controls (Figure 4A–D).

**Figure 5:**
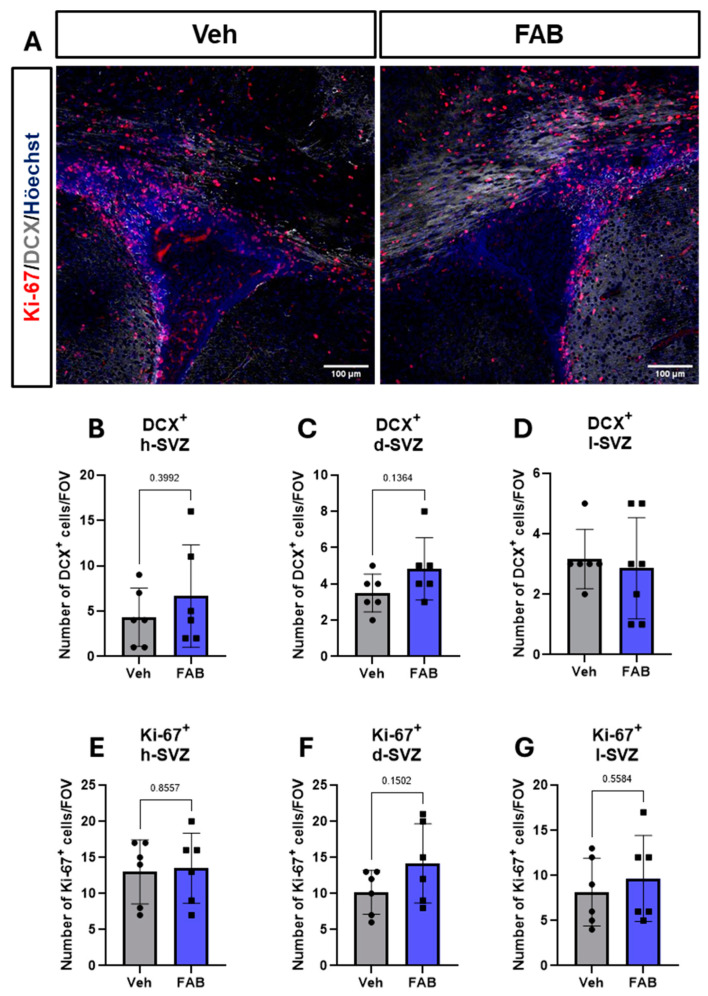
The effect of agathisflavone on proliferation of SVZ neural progenitors. Photomicrographs from agathisflavone (FAB)- and vehicle (Veh)-treated mice were acquired at the lateral ventricle regions and cell counts were performed. (**A**) Representative images of DCX-positive (grey) and Ki-67-positive (red) cells at the lateral ventricles (Scale bar: 100 μm). (**B**–**D**). Quantification of DCX-positive cells at the (**B**) horn, (**C**) dorsal, and (**D**) lateral SVZ. (**E**,**F**) Quantification of Ki-67-positive cells at the (**E**) horn (**F**) dorsal and (**G**) lateral SVZ. Data are expressed as mean of cell number per field of view ± standard deviation and tested for significance using unpaired *t*-tests.

The different microdomains of the SVZ can give rise to different neural cell types, with the dorsal SVZ and horn SVZ mainly giving rise to neurons. We, therefore, used immunostaining for the neuronal progenitor marker (DCX) together with Ki67 and the results indicate that FAB had no significant effect on NSC proliferation or neurogenesis in the SVZ at the time point analysed (Figure 5).

## 4. Discussion

Reactive gliosis is the characteristic response of astrocytes and microglia in CNS pathology. After injury of inflammatory or mechanical sources, glial cells start and coordinate a response that recruits peripheral cells and newly generated glia. However, resolution of gliosis is important to allow neurite outgrowth and function recovery [7,12,29]. In this context, flavonoids are classically known as anti-inflammatory and antioxidant compounds, and a large body of evidence has indicated they are effective in several models of CNS disease [30,31]. Flavonoids have also been shown to modulate glial phenotype which is essential to neurons in CNS pathology via regulating pathways that support their activation, proliferation, and release of neurotrophic factors toward recovery [32,33,34]. Here, we show that treatment with the flavonoid agathisflavone decreases reactive astrogliosis and microgliosis following a cerebral stab wound injury in young mice, supporting a potential therapeutic relevance for this compound in promoting repair in CNS injury.

Reactive astrogliosis is the name of the physiological reaction of astrocytes against insult of different origins [35]. This process is characterized by morphological and metabolic changes, as well as cell proliferation, which delimits the lesioned area [8,36]. Additionally, astrocytes exhibit an increase in cell size and this hyperplasia and hypertrophy is evidenced by an increase in GFAP expression [37]. In TBI, as well as in stroke and neurodegenerative models, GFAP expression is increased chronically at the lesion core and this delimits tissue recovery and repopulation by new axons [3,38]. Here, we demonstrated that FAB induces a reduction in GFAP expression at the lesioned site and this was accompanied by a reduction in cell proliferation. These data are corroborated by previous in vitro studies showing FAB can modulate astrocyte reactivity in vitro. In the scratch wound healing model in primary cultures of embryonic cortex, FAB pre-treatment decreased astrocytic process growth and a reduced GFAP expression [17]. Additionally, in a co-culture of neurons and glia, FAB treatment reduced GFAP expression of LPS-treated cultures to levels compared to the controls [19]. Together, these in vitro studies support the in vivo results from the current study and indicate that FAB reduces reactive astrogliosis, which could contribute to a faster recovery in neuroinflammatory and neuroinflammatory conditions.

In addition to astrogliosis, microglia are the resident macrophages of the CNS and, in a coordinated response with other glial cells, they compose the reactive gliosis by playing the main immune role. They are the most sensitive and rapid responders after injury and, after physical injury modelled in TBI and spinal cord injury (SCI) models, where they phagocytose debris and propagate inflammatory responses acutely at the lesions site, and subsequently play a key anti-inflammatory role in CNS repair [9,39]. However, the prolonged recruitment and proliferation of microglia at the lesion site can hamper inflammation resolution and tissue repair, thus modulating microglial activation could improve recovery [10,40]. Here, we showed that FAB inhibited microgliosis in response to a cerebral stab wound and modulated activated microglia to an anti-inflammatory phenotype associated with improved repair.

Evidence from the last 20 years has shown that microglia, for peripheral macrophages, assume different activation profiles and these are related to different phases of injury and recovery [28,41]. Some molecules expressed by activated microglia are associated with damage and the promotion of inflammation, such as MHCII, CD86, and the expression of interleukins, such as IL-12^high^ and IL-10^low^, nitric oxide synthase induced (iNOS), TNF-α, IL1-B, IFN-γ, and IL-6, in addition to nitric oxide (NO) release. In contrast, other activation profiles are more associated with debris removal and resolving the tissue injury, such as IL-4, IL-10, IL-13, Arginase1, and TGF-β, which help initiate tissue repair with the deposition of extracellular matrix [10,42].

Here, we evaluated the effects of FAB on the expression of CD16/32 and CD206 in activated microglia following cerebral stab wound in jury. These are receptors for the Fc region of immunoglobulins and a mannose receptor, which are highly expressed in pro- and anti-inflammatory microglia, respectively [43]. The results showed that FAB had no significant effect on the relative densities of CD16/32 and CD206 microglia, but did alter their relative proportions consistent with a lesion environment leaning towards repair. Consistent with this, we observed that FAB induced a ramified microglial morphological phenotype in the perilesional brain, which is related to more physiological surveillance microglia. This is supported by the effects of FAB on microgliosis in in vitro and ex vivo studies. In a co-culture of neurons and glia treated with the following pro-inflammatory stimuli: the cytokine IL-1β and the lipopolysaccharide (LPS), and FAB treatment that induced a decrease in the number of Iba-1- and BrdU-double positive cells, which indicates the number of proliferating cells. This effect was accompanied by a reduction in the number of cells expressing the pro-inflammatory transcription factor NF-κB and a down-regulation of gene expression of the pro-inflammatory cytokines TNF, IL-1β, IL-6; the chemokines CCL-2 and CCL-5; and an upregulation in the gene expression of the regulatory cytokine IL-10 [19]. A similar effect was observed in isolated microglial cultures treated with LPS, where FAB treatment reduced the number of ameboid-like microglia, a phenotype that is related to its phagocytic activity, and drastically reduced nitric oxide production. Also in these isolated cultures, FAB down-regulated the gene expression of TNF, IL-1β, IL-6, CCL-2, and CCL-5 [18].

Here, we showed that treatments with FAB exerted effects in both astrocytes and microglia, as previously shown in vitro, which suggests that FAB has a synergic effect on different cell types and/or alters the communication between these cells. Globally, our results suggest that the protective effects of FAB observed in glial cells in vitro can also be observed after mechanical injury in vivo, which could support neuronal physiology and function.

After CNS injury, tissue recovery is hampered not only by the prolonging of glial scarring, but also by a limited level of neurogenesis and tissue repopulation, which contributes to tissue loss of function. In the adult mammal brain, neurogenesis is restricted to the subventricular zones (SVZ) and to the hippocampus dentate gyrus [44,45]. Neurogenesis stimulation and neural differentiation in these areas may be an alternative for treating brain diseases. Hence, here we examined the capacity of FAB to modulate neurogenic populations in the SVZ niche as potential synergic protective effect to the modulation of reactive gliosis. Our results showed that, compared to control animals, FAB increases the number of GFAP-positive cells tracked using the GFEC reporter mouse line [24] at all SVZ subdomains. The GFAP-positive cells at the SVZ are considered neural stem cells (type B cells) that give rise mainly to neurons, although in the dorsal SVZ, they give rise to oligodendrocyte precursor cells (OPCs) that migrate to white matter [26,46,47]. The results of the present study also indicated that FAB induced a trend toward increasing the number of DCX-positive cells in the d-SVZ, a region where neuroblasts predominantly give rise to neurons that migrate to the olfactory bulb [45]. Our findings are consistent with previous studies showing FAB-induced neuronal differentiation of mouse embryoid bodies derived from pluripotent stem cells and presented a synergistic effect with retinoic acid [23]. In another study, in a co-culture with neurons and glial cells, agathisflavone treatments have been shown to increase the number of β-tubulin III- and DCX-positive cells after seventy-two hours, and that this effect was mediated by oestrogen receptors [21]. Additionally, in the scratch wound model, besides from decreasing astrogliosis, FAB treatments increased the number of neuronal processes that entered the scratched area and the gene expression of the neurotrophic growth factor NGF and glial derived growth factor GNDF [17]. Together, these studies support a pro-neurogenic action of FAB, although our current study was limited to observing the effects of FAB at a single time point, and fate mapping studies over the longer term are required to determine whether FAB promotes the generation of neuroblasts and neurons and mechanisms involved in neurogenesis and in regulation of glial cells response.

## 5. Conclusions

Taken together, our results demonstrate that agathisflavone (FAB) modulates reactive gliosis and promotes neuroprotective responses following traumatic brain injury (TBI) in a murine stab wound model. We demonstrated that FAB treatment significantly attenuated astrocyte and microglial reactivity at the lesion site, evidenced by reduced GFAP expression and decreased Iba1+ microglial density in both lesion and perilesional sites. Furthermore, FAB promoted a shift in microglial activation towards a reparative, anti-inflammatory CD206+ phenotype, as indicated by an increased CD206+/CD16/32+ ratio. These protective effects accompanied an increase in the neuroblast neurogenic population at the SVZ, without significantly altering the doublecortin (DCX) neuronal progenitor population at the evaluated timepoint. These findings suggest that FAB facilitates a neuroprotective environment by tempering gliosis and inflammation while supporting neurogenesis at the SVZ, highlighting its therapeutic potential in promoting recovery after CNS injury. Future studies should explore the long-term effects of FAB on neurogenesis and functional recovery using fate-mapping, as well as its mechanisms of action in modulating glial and neural cell dynamics. Together, these findings support the development of FAB as a promising candidate for CNS repair therapies.

## Data Availability

Data are contained within the article.
